# Development of Monetary and Social Reward Processes

**DOI:** 10.1038/s41598-017-11558-6

**Published:** 2017-09-11

**Authors:** Di Wang, Tongran Liu, Jiannong Shi

**Affiliations:** 1 CAS Key Laboratory of Behavioral Science, Institute of Psychology, Chinese Academy of Sciences, Beijing, China; 2 Department of Psychology, University of Chinese Academy of Sciences, Beijing, China; 3 Department of Learning and Philosophy, Aalborg University, Copenhagen, Denmark

## Abstract

The current study investigated monetary and social reward processing in children, adolescents and adults with adapted incentive-delay tasks and self-report questionnaires. Both tasks had three levels of reward magnitudes (no, low, and high). Qualified participants received 15 Chinese Yuan and an honor certificate as monetary and social rewards, respectively. The results indicated that both monetary and social rewards effectively speeded up responses for all three age groups as reward magnitude increased in the choice reaction time task. Among adolescents and adults, males exhibited faster responses in high reward than in low reward condition, while females responded equally fast in both conditions. Among children, girls responded faster to high reward than low reward condition. However, boys committed more errors than girls in low and high reward conditions, and they had exhibited more errors in high reward than that in no reward condition for social reward. Regarding the subjective ratings, both children and adolescents reported higher motivation for social reward than for monetary reward. These findings indicated that the males in the adolescent and adult groups were more sensitive to reward than were the females. Moreover, tangible and quantitative social reward had stronger incentive power than monetary reward among children and adolescents.

## Introduction

Rewards can affect individual’s learning and behavior and function to maximize benefits. An individual’s motivation is highly active when processing reward-related information^1^. The development of reward preference begins at birth and continues throughout an individual’s life in conjunction with the development of the brain reward system, such as the striatum, nucleus accumbens, and prefrontal cortex (PFC)^2–6^. The degree of reward sensitivity has been found to be related to alcohol and drug abuse behaviors in children and adolescents^7^. Therefore, it is vital to study developmental changes in reward processes and how rewards affect children and adolescents’ behavior and learning.

Rewards such as food, monetary and social rewards are generally considered to be related to human motivation and behavior^8–11^. Among these rewards, social and monetary rewards are widely studied and regarded as two essential and advanced reward processes that have large impact on an individual’s behavioral development. Social rewards such as smiling faces, encouraging gestures and verbal praise can be considered as positive reinforcers that increase the likelihood that the corresponding behavior will be executed in the future^9, 10, 12^. Meanwhile, it has been widely reported that goal-directed behavior is also well-organized and the brain reward system is highly active when a person is anticipating monetary reward^13–15^. Furthermore, monetary incentive delay (MID) and social incentive delay (SID) tasks are classical paradigms that have frequently been used to investigate monetary and social reward processing^10, 16–18^. Despite some alterations to the paradigms according to different research requirements, reward anticipation, target response and reward feedback delivery are the three basic stages in MID and SID tasks^10, 18^.

Using the MID and SID task paradigms, recent studies on monetary and social rewards have endeavored to identify their incentive differences^9, 10, 16, 17, 19^. Most of these studies have involved adult participants, and the results indicated that monetary reward had stronger incentive value than social reward not only at the behavioral level but also on reward-related cortex activation^10, 19–21^. However, developmental studies have shown that the incentive value of social reward is no less than that of monetary reward in children and adolescents^16, 17^, which might reveal the differences in reward processes in children and adolescents compared to adults. Based on their less developed brain reward system and limited cognitive ability, children have been found to be more sensitive to social reward than object reward at as early as 6 years old^12^; the concept of money is not fully established until children reach 8 years old^22, 23^. Therefore, during childhood, social reward functions as effective reinforcers while monetary reward does not yet exert strong incentive power. Adolescents undergo a period during which important structural and functional refinements occur in the brain reward system^4, 5, 24–26^. Compared with children and adults, the ventral striatum of adolescents is over-responsive to expectation of both extrinsic and intrinsic rewards^27, 28^,while the PFC, which is responsible for executive function, is still developing^4^. Subjectively, both monetary and social rewards are attractive to adolescents^5, 9, 16, 17, 29^.

The aim of the current study was to investigate how monetary and social reward processes affect individuals’ cognitive performances from the developmental perspective. We enrolled three age groups—children, adolescents and adults—and compared their performances on MID and SID tasks. The studies of Demurie (2011, 2012) were typical in that they used MID and SID tasks to compare the incentive power of monetary and social reward for participants between 8 and 16 years old. In their studies, to control participants’ motivation towards different reward magnitudes and compare only their motivation towards different reward types, points (0, 5 and 15 points) were added to compliments and pictograms as social reward feedback in the SID task to align with the 0, 5 and 15 eurocents the participants earned as monetary reward feedback in the MID task. They found that response time (RT) decreased with each increase in the anticipated reward. Inspired by this design^16, 17^, in our research, fast and accurate responses earned the participants 0, 1 or 2 points for both monetary and social rewards. In this way, the monetary and social rewards were more comparable. In addition to the manipulation of quantification, our study involved three novel attempts compared with Demurie’s studies. First, in Demurie *et al*.’s studies^16, 17^, accurate responses faster than 500 ms earned participants the corresponding reward. Given the large age range in our study, the difficulty of earning a reward varied for children, adolescents and adults. Thus, in our study, each participant’s baseline RT was measured before the formal experiment and set as the reward standard. It has been verified in the previous research that using each participant’s mean RT as a baseline could effectively incentive the participant’s motivation for the reward stimuli^15^. Second, in Demurie’s studies^16, 17^, all participants received the same amount of money at the end of the experiment regardless of their performances in the tasks. Our research aimed to motivate the participants to earn their monetary and social rewards by relating their task performances to the ultimate received rewards. Specifically, only an actual obtained reward score above 60% of the total score earned the participants the corresponding monetary and social rewards in the MID and SID tasks. Third, in Demurie’s studies, only the smiling faces were presented as feedback stimuli immediately at the end of trials as social reward. Compared with the monetary reward, the social reward was intangible, non-accumulative and had only short-term effects. In our study, in addition to smiling face feedback, an honor certificate was issued to qualified participants following the SID task.

Our hypotheses were as follows: (1) Both monetary and social rewards will enhance performance for all three groups of participants. The incentive power of social reward will exceed that of monetary reward for children and adolescents, while monetary reward will have a stronger incentive value for adults. (2) Regarding subjective ratings, the motivation for social reward will be similar to the motivation for monetary reward in children and adolescents, while adults will exhibit higher motivation for monetary reward.

## Results

### Reaction time

**Figure 1:**
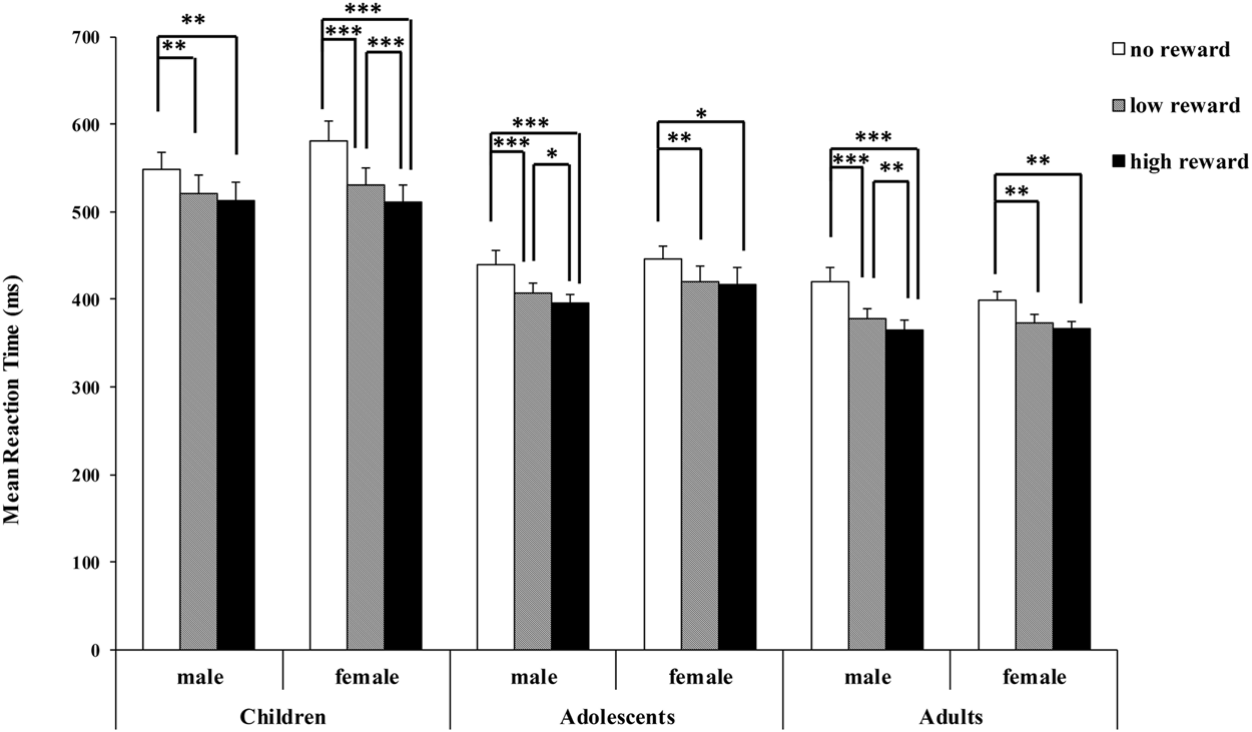
Mean reaction times (ms) as a function of age group, participant gender and reward magnitude in the formal experiment.

The mean RTs of the correct responses for each task are displayed in Fig. 1. The mean RTs, response error rates and subjective ratings are reported in Table 1.

**Table 1:** Mean and SD of RT (ms), error rate (%)for male and female in each group.

			MID task			SID task		
			no reward	low reward	high reward	no reward	low reward	high reward
Children	male	RTs (SD)	559.04 (80.09)	529.05 (83.19)	515.34 (88.57)	537.65 (74.13)	512.17 (84.06)	511.01 (80.24)
		Error rates (SD)	9.67 (5.43)	11.00 (5.11)	10.89 (6.39)	8.56 (6.07)	11.11 (5.73)	14.00 (7.61)
	female	RTs (SD)	583.35(107.15)	528.17 (77.19)	508.32 (69.40)	577.69 (84.92)	531.56 (83.83)	514.17 (78.05)
		Error rates (SD)	9.11 (8.26)	6.89 (3.93)	8.44 (7.44)	8.22 (4.06)	8.11 (4.75)	6.44 (4.71)
Adolescents	male	RTs (SD)	439.36 (59.87)	406.43 (44.84)	399.94 (46.84)	440.89 (61.84)	407.87 (51.18)	390.63 (41.82)
		Error rates (SD)	9.00 (6.10)	6.89 (4.87)	6.89 (4.58)	8.56 (7.21)	8.33 (5.63)	7.33 (4.36)
	female	RTs (SD)	446.86 (58.02)	423.81 (65.04)	420.75 (70.27)	445.08 (60.65)	417.26 (76.01)	414.46 (75.08)
		Error rates (SD)	5.89 (4.67)	5.56 (4.02)	4.44 (4.07)	5.44 (3.91)	5.67 (5.48)	7.11 (4.39)
Adults	male	RTs (SD)	424.28 (69.28)	380.07(42.21)	364.16 (36.93)	417.13 (64.22)	375.94 (44.86)	365.83 (47.55)
		Error rates (SD)	7.56 (6.33)	5.22 (4.40)	6.00 (4.53)	6.56 (5.02)	6.00 (4.07)	5.33 (3.99)
	female	RTs (SD)	405.95 (42.26)	376.04 (36.06)	368.26 (35.05)	391.62 (45.16)	371.57 (33.51)	364.15 (32.18)
		Error rates (SD)	4.44 (4.48)	2.78 (2.49)	4.67 (4.42)	3.89 (3.19)	4.00 (3.38)	4.33 (3.77)

The results revealed a main effect of reward magnitude, *F*(2,168) = 113.19, *p* < 0.001, *η* ^2^ = 0.57; post hoc comparisons revealed significantly faster RTs for the high-reward than for the no-reward condition (*t* (89) = −11.48, *p* < 0.001), for the low-reward than for the no-reward condition (*t* (89) = −10.32, *p* < 0.001) and for the high-reward than for the low-reward condition (*t* (89) = −6.21, *p* < 0.001). In addition, a main effect of age group, *F* (2, 84) = 50.81, *p* < 0.001, *η* ^2^ = 0.55, was observed; the post-hoc comparisons revealed significantly faster RTs for the adults (*t* (58) = −9.68, *p* < 0.001) and the adolescents (*t* (58) = −7.27, *p* < 0.001) than for the children. The difference in RTs between the adolescents and the adults was marginally significant (*t* (58) = −2.41, *p* = 0.06).

There was a three-way interaction among reward magnitude, age group and participant gender, *F* (4,168) = 4.50, *p* = 0.002 < 0.01, *η* ^2^ = 0.10. Post hoc comparisons revealed that within the children’s group, boys reacted faster under the high-reward than the no-reward condition (*t* (14) = −3.72, *p* = 0.001 < 0.01) and under the low-reward than the no-reward condition (*t* (14) = −3.43, *p* = 0.003 < 0.01). However, the difference between the low-reward and the high-reward condition was not significant (*t* (14) = −1.84, *p* = 0.21). Girls in the children’s group reacted faster in the high-reward than in the no-reward condition (*t* (14) = −7.32, *p* < 0.001) and in the low-reward than in no-reward condition (*t* (14) = −6.26, *p* < 0.001). Furthermore, girls reacted faster in the high-reward than in the low-reward condition (*t* (14) = −4.61, *p* < 0.001).

Among the adolescents, males reacted faster under the high-reward than the no-reward condition (*t* (14) = −4.74, *p* < 0.001), faster under the low-reward than the no-reward condition (*t* (14) = −4.08, *p* < 0.001), and faster under the high-reward than the low-reward condition (*t* (14) = −2.94, *p* = 0.013 < 0.05). The female adolescents reacted faster under the high-reward than the no-reward condition (*t* (14) = −3.00, *p* = 0.011 < 0.05), and faster under the low-reward than the no-reward condition (*t* (14) = −3.14, *p* = 0.007 < 0.01). However, the difference in RTs between the high-reward and low-reward conditions was not significant for the female adolescents (*t* (14) = −0.73, *p* = 1.00).

In the adult group, males reacted faster under the high-reward than the no-reward condition (*t* (14) = −5.89, *p* < 0.001), faster under the low-reward than the no-reward condition (*t* (14) = −5.28, *p* < 0.001), and faster under the high-reward than the low-reward condition (*t* (14) = −3.22, *p* = 0.005 < 0.01). Female adults reacted faster under the high-reward than the no-reward condition (*t* (14) = −3.44, *p* = 0.003 < 0.01), and faster under the low-reward than the no-reward condition (*t* (14) = −3.09, *p* = 0.008 < 0.01). However, the difference in RTs between the high-reward and low-reward conditions was not significant for the female adults (*t* (14) = −1.88, *p* = 0.19).

To further analyze the performances of the participants who won both monetary and social rewards, the mean RTs of the first part (trials before reaching 108 points) and the second part (trials after reaching 108 points) were calculated for each reward type. After the 108-point threshold the average number of trials per participant was more than 30 trials. This result indicated that there were sufficient datapoints for each participant after splitting the dataset into before/after 108 points. The results revealed a main effect of age group, *F*(2,60) = 36.88, *p* < 0.001, *η* ^2^ = 0.55, and post hoc comparisons revealed significantly faster RTs for the adults (386 ms) (*t* (45) = −8.43, *p* < 0.001) and the adolescents (418 ms) (*t* (35) = −6.12, *p* < 0.001) than for the children (539 ms). The difference in RTs between the adolescents and the adults was not significant (*t* (46) = 1.76, *p* = 0.25). The part type (before or after the 108-point threshold) factor interacted significantly with age group, *F*(2,60) = 5.27, *p* = 0.008 < 0.01, *η* ^2^ = 0.15. Post hoc comparisons revealed that the RTs of adolescents were significantly slower after reaching 108 points (426 ms) than before reaching 108 points (409 ms) (*t* (18) = 3.55, *p* = 0.001 < 0.01). The mean RT differences between the first and second parts were not significant for children (*t* (17) = −0.16, *p* = 0.88) and adults (*t* (28) = −0.47, *p* = 0.64).

### Error rate

**Figure 2:**
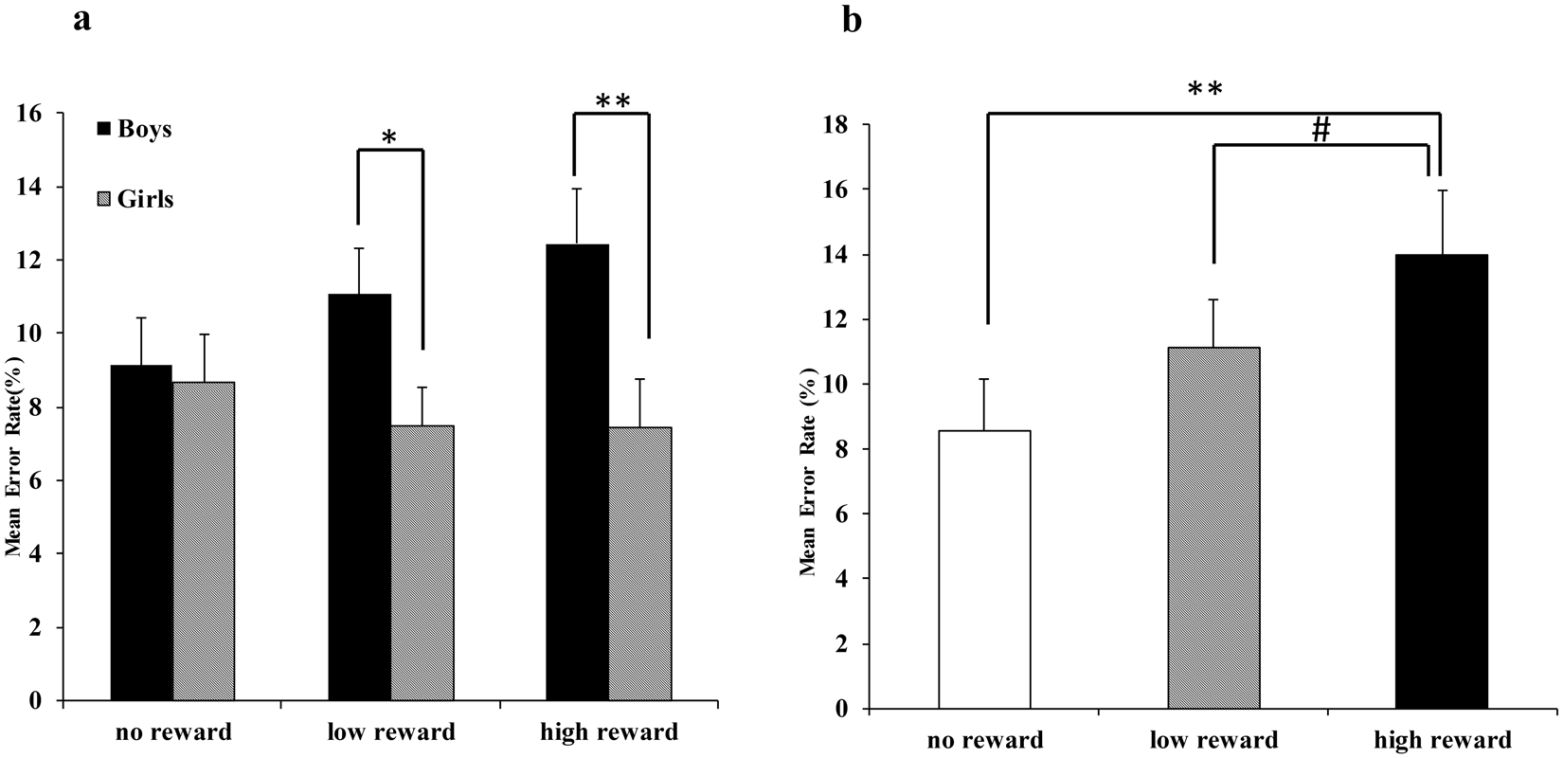
(**a**) Mean error rates as a function of reward magnitude and participant gender for the children’s group. (**b**) Mean error rates as a function of reward magnitude for boys in the children’s group on the SID task.

Participants’ error rates are presented in Fig. 2. The results revealed a main effect of age group, *F* (2, 84) = 9.85, *p* < 0.001, *η* ^2^ = 0.19, post hoc comparisons revealed that the average error rate was lower for the adults (*t* (58) = −4.3, *p* < 0.001) and the adolescents (*t* (58) = −2.6, *p* = 0.027 < 0.05) than for the children. The difference between the adults and the adolescents was not significant (*t* (58) = −1.7, *p* = 0.26). In addition, a main effect of gender, (*F*(1, 84) = 9.14, *p* = 0.003 < 0.01, *η* ^2^ = 0.10) was observed, and males committed more errors than females.

There was a three-way interaction among reward magnitude, age group and participant gender, *F* (4,168) = 3.57, *p* = 0.008 < 0.01, *η* ^2^ = 0.08. Post hoc comparisons revealed that in the children’s group, the error rates for boys were higher than those for girls when the reward magnitudes were low (*t* (28) = 2.4, *p* = 0.018 < 0.05) or high (*t* (28) = 3.13, *p* = 0.002 < 0.01). Under the no-reward condition, the error rate difference between the boys and girls was not significant (*t* (28) = 0.24, *p* = 0.80).

Moreover, there was a four-way interaction among reward type, reward magnitude, age group and gender, *F*(4,168) = 2.45, *p* = 0.048 < 0.05, *η* ^2^ = 0.06. Post hoc comparisons revealed that in the children’s group, for the social reward, boys committed more errors when the reward magnitude was high than when there was no reward (*t* (14) = 3.38, *p* = 0.002 < 0.01) and committed marginally more errors under the high reward condition than under the low reward condition (*t* (14) = 2.42, *p* = 0.051).

A further error rate analysis of participants who won both monetary reward and social reward revealed only a main effect of age group, *F* (2,60) = 10.01, *p* < 0.001, *η* ^2^ = 0.25. Post hoc comparisons revealed that the error rate of adults (4.6%) was lower than that of children (9.9%) (*t* (45) = −4.42, *p* < 0.001). Error rate differences between children and adolescents (7.0%) (*t* (35) = 2.23, *p* = 0.09) and adolescents and adults (*t* (46) = 2, *p* = 0.14) were not significant.

### Subjective rating

**Figure 3:**
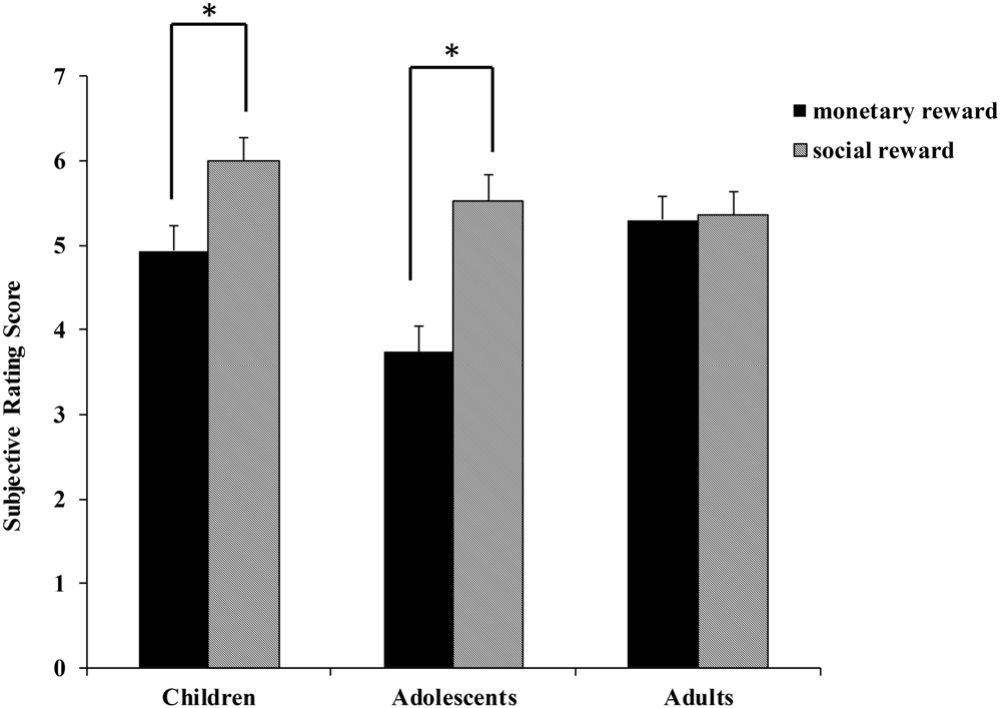
Subjective rating scores as a function of age group and reward type.

The subjective rating scores are shown in Fig. 3. The results revealed a main effect of reward type (*F*(1,84) = 15.87, *p* < 0.001, *η* ^2^ = 0.16): the participants rated social reward higher than monetary reward. A main effect of age group was found (*F* (2, 84) = 4.89, *p* = 0.01 < 0.05, *η* ^2^ = 0.104) and the adolescents rated the reward stimuli lower than did the children (*t* (58) = −2.91, *p* = 0.014 < 0.05) and the adults (*t* (58) = −2.45, *p* = 0.05). The subjective rating difference between the children and the adults was not significant (*t* (58) = 0.47, *p* = 1.00).

The reward type factor interacted significantly with age group, *F*(2, 84) = 4.19, *p* = 0.018 < 0.05, *η* ^2^ = 0.091. Post hoc comparisons revealed that the children (*t* (29) = −2.51, *p* = 0.014 < 0.05) and the adolescents (*t* (29) = −4.24, *p* < 0.001) rated monetary reward lower than social reward. Among the adults, the difference in the subjective ratings between monetary and social rewards was not significant (*t* (29) = −0.16, *p* = 0.88).

## Discussion

The current study investigated the features of monetary and social reward processing from the developmental perspective. By using the adapted MID and SID tasks and self-reporting scales for the two types of rewards, both objective performance measures and subjective ratings could be considered simultaneously to investigate the development of reward processes in children, adolescents and adults.

Similar to previous research, both monetary and social rewards effectively motivated behavior across the three age groups. The motivation was reflected in the faster response speeds in the reward-related trials than in the no-reward trials^9, 10, 12, 15, 17^. The participants’ faster response speeds under reward conditions indicated that the two types of rewards raised motivation and stimulated more attention resource allocation in all three groups^15, 30–33^. In addition to the simple effect of reward, faster response speeds were observed with the increment of reward magnitude across the three groups. In previous studies^10, 17^, when smiling faces of different intensities were presented as low and high social reward feedback, the difference in response speeds between low and high reward conditions was not significant. However, the SID-plus task used in Demurie *et al*.’s study^17^ and our current research, which combined low and high rewards with different points, revealed differences in response speeds between the low and high reward conditions. This finding suggests that through the quantification of reward, reward magnitude regulated the behavior of the participants more efficiently. That may indicate that quantification of reward is an effective way to help participants discriminate different reward magnitudes and then regulate their response speeds accordingly. Meanwhile, this result demonstrated that all the participants, including the children, understood the rules of the experiment well.

Regarding the RT results, as soon as the cumulative score reached 108 points, only the adolescents’ response speeds decreased. This indicated that the adolescents’ behavior was finely adjusted by reward—not only the anticipation of reward but also the achievement of the ultimate reward standard. It was also found that adolescents were more sensitive to reward than were children and adults. This finding is consistent with previous findings, which indicated that compared with children and adults, the behavioral changes of adolescents are driven much more by rewards, including both monetary and social rewards^24, 25, 28^.

In the current study, the sensitivity to reward magnitude reflected on RT in two ways. First, the participants responded to the targets faster in the low and high reward trials than in no reward trials. Second, they responded to the targets faster in high reward trials than in low reward trials. All three age groups in the current study were sensitive to reward magnitude in the first way. Regarding the second way, in the adolescent and adult groups, males exhibited faster response speed in the high-reward condition than in the low-reward condition. In contrast, females in the two groups reacted equally to those two reward conditions. Reward magnitude did not affect the behavior of females as strongly as it did for males in adolescent and adult groups. Therefore, it can be speculated that males in the two groups were more sensitive to the different reward magnitudes than females. The greater sensitivity resulted in effective and immediate behavior regulation based on the different reward magnitudes. This result may be due to males are more sensitive to reward than are females during adolescence^34^ and adulthood^10, 35, 36^. First, from the evolutionary perspective, across many species (including our own) males are responsible for searching for reward stimuli such as food and water to produce offspring and obtain more territory^37^. This responsibility and long-term practice may have made males more sensitive to reward. Second, the Behavioral Approach System (BAS) is a brain system associated with dopaminergic activity and response for mediating responses to conditioned and unconditioned reward stimuli, while the Behavioral Inhibition System (BIS) inhibits inappropriate behavior^38^. Evidence from studies using BAS/BIS scales indicates that among adolescents and adults, males exhibit higher levels of sensation seeking behaviors and lower levels of impulse control than do females, while females are associated with decreased sensitivity to rewards and increased sensitivity to punishment^38, 39^. Third, the modeling of the incentive processing system was tied to puberty^40, 41^. Specifically, studies have found that among adolescents and young adults, higher levels of testosterone predicted greater activation of the brain regions sensitive to reward during reward trials^42^. Therefore, from adolescence to adulthood, men are considered to be more sensitive to reward than women. Reward valence and reward magnitude are two fundamental features of reward processes^43^. Reinforcement sensitivity theory indicates that individuals with stronger sensitivity easily detect reward cues and establish faster associations between instrumental responses and rewards^44^. Specifically, in the current study, more sensitivity to reward was reflected in more sensitivity to reward magnitude.

Interestingly, girls in the children group responded faster in the high reward condition than in the low reward condition; however, for boys, differences in response speeds between the low and high reward conditions were not significant. The brain reward system is less developed for children and does not reach functional maturity until puberty^40, 41^. Therefore, the gender effect in the children’s group was not the same as that observed in the other two age groups. Studies employing Stroop and Go/Nogo tasks have indicated that the executive function of girls is better than that of boys on many dimensions during childhood, especially inhibition control^45–47^. The difference may be because boys’ and girls’ brains develop and mature at different rates during childhood^48^. Specifically, the development of brain gray matter volume, which is closely related to executive function, peaks at the age of 8.5 years for girls and 10.5 years for boys^48^. Furthermore, development of the frontal lobe, which is critical in executive function, occurs faster in girls than in boys^49^. It could be deduced that girls’ better executive functions helped them to regulate their behavior according to the potential reward magnitudes, especially under the low and high reward conditions. Moreover, reward motivates top-down attention to energize goal-directed behavior^33, 50^. External rewards and individuals’ executive function interact to affect behavior^50–53^. As proposed by Sonuga-Barke and Sergeant^54^, immature executive function was more apparent in situations in which affective and motivational processes interacted with executive function. Specifically in the current study, when the reward magnitudes were low and high, boys committed more errors than girls, and boys committed more errors in the high reward trials than in the low and no reward trials for the social reward. Judging from the subjective ratings of motivation for the two types of rewards, children considered the social reward more rewarding than the monetary reward. Typically, compared with low rewards, high reward help people limit failure to act on current goals and inhibit improper behavior^55^. However, the current study showed that high motivation and high reward magnitude had a negative effect on boys’ performance. Moreover, this result also demonstrated that participants could be aware of the reward condition (monetary or social) when performing the corresponding tasks, instead of just focusing on the accumulation of the scores.

Regarding the subjective ratings of motivation for monetary and social rewards, and inconsistent with previous research on children and adolescents^9^, motivation for social reward was reported higher than that for monetary reward among the children and adolescents in our study. In Kohls *et al*.’s study^9^, only smiling faces were presented as social reward and were non-accumulative. In the current study, it is noteworthy that in addition to using cartoon smiling faces as social reward feedback stimuli, an honor certificate was awarded as a social reward. It reported how many smilling faces the participant had got in the SID task and was regarded as material carrier of social reward. In school education, the honor certificate conveys the approval from teachers and respect from the peers. Therefore, it is a rare example of tangible social reward. During childhood and adolescence, evaluation from adults, especially teachers and parents, is considered much more important than any other type of incentive^56^. Unlike the immediately available cartoon smiling faces, which have only a short-term effect, honor certificates have longer-lasting effects and are more memorable^9^. Furthermore, adolescents and children are financially dependent on their parents and control little money independently^57^. Therefore, an honor certificate has an advantage for motivating behavior among children and adolescents. Although the adolescents and children in this study subjectively reported higher motivation for social reward than for monetary reward, consistent with previous research^17, 58^, this reward preference was not reflected in their faster RT in the SID task. This may be because the incentive value of the social reward was reduced by repeated presentation^17^. Moreover, as long as their points exceeded 108 (60% of the possible total score), the participants received the social and monetary rewards. After reaching the reward standard, the motivation of the participants decreased rapidly, especially the adolescent group. For these reasons, the subjective ratings were not completely reflected in the participants’ behavior.

In future studies, the ultimate social and monetary rewards could be entirely dependent upon the behavior of the participants in the corresponding task. Thus, the higher scores participants achieve on a task, the higher the magnitude of the reward they would ultimately receive. This approach is likely a better way to investigate motivation for monetary and social rewards. In addition to the extrinsic rewards, cognitive feedback such as the “√” and “ × ” used in our study (see Methods) and successful performances in the tasks could also be regarded as intrinsic rewards that could also impact behavior^27, 59, 60^. They share similar neural substrates with external rewards, such as the dopaminergic system, nucleus accumbens and ventral striatum^27, 59^. Satterthwaite and his colleagues (2012) revealed that in response to external rewards and intrinsic rewards, the VS experiences similar “inverted-U” curves as the individual grows. For internal rewards, VS activation peaks at approximately 16 years of age. Further study could endeavor to discriminate between the effects of intrinsic and external rewards from an individual development perspective, 16-year-old adolescents whose VS activation is peaking for external and internal rewards could be recruited as focus group.

The results suggest the following implications. First, during childhood, the gender differences which are reflected in responding to reward magnitude may be mainly influenced by developmental differences in executive function, whereas during adolescence and adulthood, the performance differences regarding reward magnitude between males and females may be mainly affected by differences in reward sensitivity. Second, our study’s use of an honor certificate to represent social reward was a novel approach. Thus, the incentive value of the social reward was comparable to that of the monetary reward. Third, the children and adolescents subjectively rated the tangible social reward based on their good performances as more rewarding than money. This finding has implications for teachers and parents because it suggests that in addition to smiling faces and verbal compliments, tangible social rewards such as honor certificates can be employed as effective social rewards. Fourth, too-high motivation may have negative effects on the behavior of boys. Therefore, moderate motivation is more suitable for boys during childhood.

In conclusion, both monetary and social rewards can effectively enhance behavioral performances in children, adolescents and adults. Compared with children and adults, adolescents were more sensitive to reward. Among the children in this study, girls regulated their behavior according to low and high reward magnitudes, but boys were not as sensitive to reward magnitude as were girls. Additionally, boys committed more errors when the reward magnitudes were low or high. Among the participating adolescents and adults, males modulated their behavior according to low and high reward magnitudes, whereas females did not. The children and adolescents exhibited higher motivation for social reward, while adults rated monetary and social reward equally.

## Methods

### Ethics

The study was approved by the Institutional Review Board of the Institute of Psychology, University of Chinese Academy of Sciences. It was carried out in accordance with the APA ethical standards for the treatment of subjects.

### Participants

Three groups of participants were enrolled, 30 children (7.9–8.5 years old, M = 8.3, SD = 0.2), 30 adolescents (12.9–13.5 years old, M = 13.2, SD = 0.2) and 30 adults (20.1–29.4 years old, M = 24.3, SD = 2.5). The gender ratio was 1:1 within each group. The adults were undergraduate and graduate students in a university, and they provided written informed consent prior to the experiment. The children and adolescents were recruited from a local primary school and middle school, and their parents provided written informed consent for their participation. Specific informed consent has also been obtained from each participant to publish the information or images that might lead to identification of the participant in an online open-access publication. All the participants were right-handed with normal or corrected-to-normal vision and were free from neurological or psychiatric disorders.

### Procedures

The presentation of stimuli and recording of RTs and error rates were controlled by E-Prime 2.0 software (Psychology Software Tools, Pittsburgh, PA). The entire experiment was divided into three stages: baseline measurement of RT, formal experiments regarding the monetary and social rewards, and post-experiment subjective ratings concerning motivation for the two types of rewards. Participants were asked to answer several questions before the formal experiment to make sure that they fully understood the tasks.

### Baseline RT measurement

Participants were unaware of the goal or the design of the experiments. They were seated in a dimly lit and sound-attenuated room and instructed to respond to the shape of a target figure (triangle or square). They were explicitly informed of the correct response button for each figure before the experiment. At the beginning of each trial, a white fixation cross (0.48° × 0.48° in visual angle) appeared at the center of a black screen for 600–1000 ms. Next, the target figure (square: 2.12° × 2.12° or equilateral triangle: 3.22° × 2.79°) was presented in the center of the screen for 500 ms. The participants were instructed to respond to the target shape as quickly and accurately as possible upon the presentation of the target stimuli using one of two response buttons (the left or right button on the computer mouse) under the right index and middle fingers. After the target figure presentation, the fixation cross was presented again for 1400–1800 ms, followed by the feedback stimulus (a “√” for a correct response or an “×” for an incorrect response) for 500 ms. This stage included 40 trials. Through this process, the participants became familiar with the task. Subsequently, the average RT for each participant was used as that participant’s baseline RT in the next stage.

### Formal experiment

In this stage, participants were informed that the current study was about reward. Fifteen Chinese Yuan or an honor certificate was awarded to qualified participants as the monetary or social reward, respectively. Commonly, honor certificates are records of praise from other people, especially from teachers and superiors. “Congratulations for achieving XX social approval points (the total number of smiling faces which were obtained in the formal experiment, ranged from 108–180 points) in the psychological experiment!” was printed on the certificate to denote the social reward.

**Figure 4:**
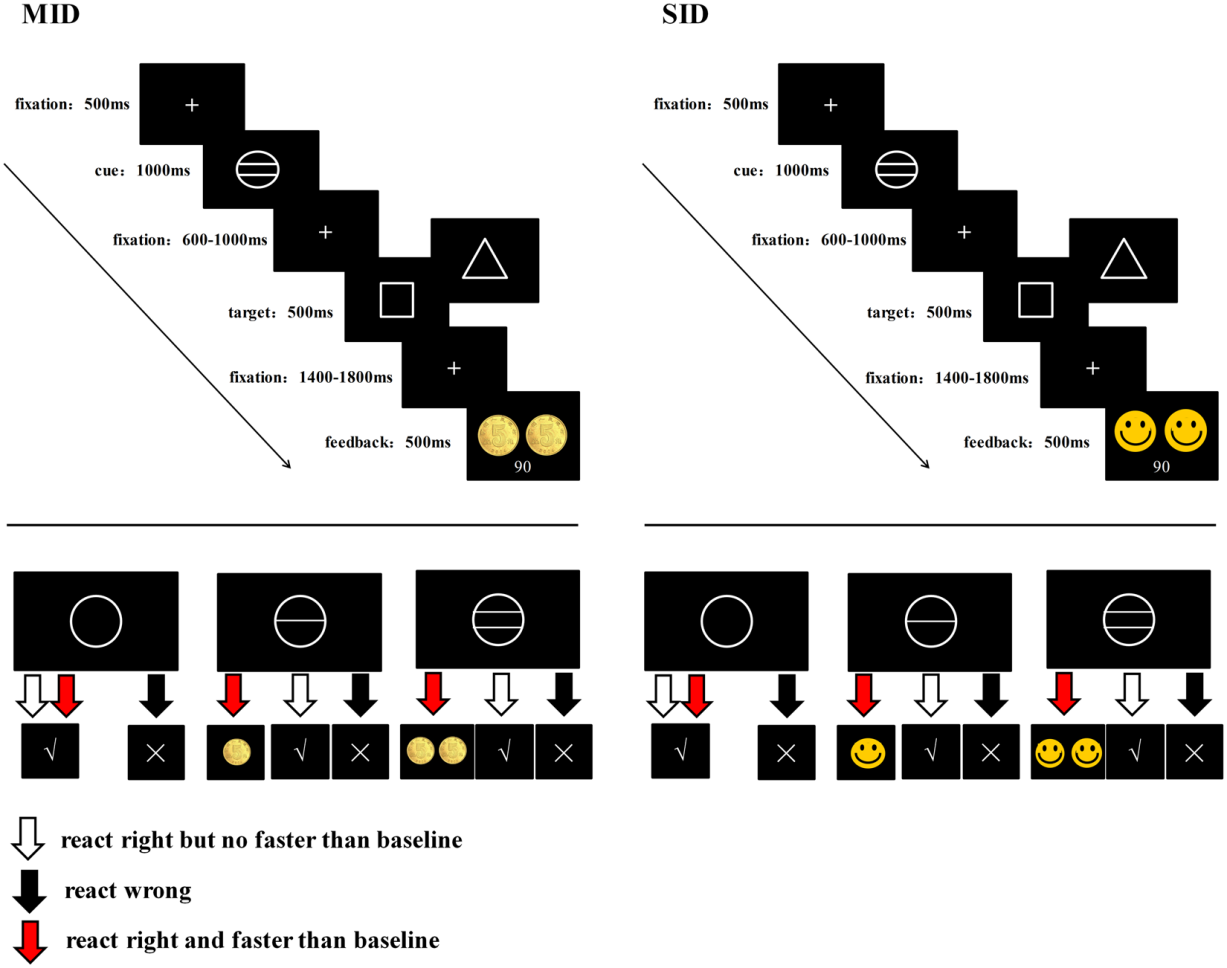
Examples of the trial sequence in the MID and SID tasks. Participants were asked to discriminate the shape of the target figure (triangle or square). During the reward anticipation period, a hollow circle or a circle with one or two horizontal lines signaled the magnitude of the potential reward in the current trial. Based on the task, the reward magnitude of the current trial and the participant’s response, Chinese coins or smiling faces were presented as monetary or social reward feedback stimuli.

Then, participants were introduced the experiment instruction while looking at the corresponding trial sequence pictures. Through practice, it was a good way to help participants understand the rules of the experiment, especially the children. The experiment consisted of two tasks: the adapted MID and SID tasks^14, 16–18^ (Fig. 4). The task sequence was counterbalanced across the participants in each group. Each task consisted of 180 trials in three continuous blocks. Reward magnitudes of no reward, low reward and high reward accounted for a third of each block and were presented randomly.

At the beginning of each trial, a white fixation cross (0.48° × 0.48°) appeared at the center of a black screen for 500 ms, followed by a cue (2.07° × 2.07°) for 1000 ms. The magnitude of the potential reward varied on three levels as indicated by the number of horizontal lines in the cue. An empty white circle indicated no reward, while a white circle with 1 or 2 lines indicated that the reward magnitude was low or high, respectively. After a variable cue-target interval of 600–1000 ms, the target feature (a square: 2.12° × 2.12°or a triangle: 3.22° × 2.79°) was presented in the center of the screen for 500 ms. As in the baseline measurement stage, the participants were instructed to respond to the shape of the target. The response button for each figure (triangle or square) in the formal experiment was consistent with the buttons used in the baseline measurement stage.

Based on the task, the reward magnitude of the current trial and the participant’s response, feedback stimuli were presented for 500 ms after a variable target-feedback interval of 1400–1800 ms. For no-reward trials, a “√” or “×” was presented as the feedback stimuli according to whether the correct button was pressed. In low-reward trials, one 0.5 Chinese Yuan coin (3.88° × 3.88°) or a smiling cartoon face of the same size was presented as monetary or social low-reward feedback stimuli when the participant provided accurate responses that were faster than their baseline RT. Similarly, accurate and faster RTs earned the participants two 0.5 Chinese Yuan coins (8.01° × 3.88°) or two smiling cartoon faces as feedback under the high-reward condition. Meanwhile, for the low and high feedback stimuli, one and two points, respectively, were added to the total points for the corresponding reward type. Slower RTs or incorrect answers in the low and high reward trials received only a “√” or “×” but no additional points. The cumulative points were presented under the reward feedback stimuli to ensure that the participants were aware of their performances in the current task. After completing an MID or SID task, the total points that the participants had earned for that reward type were presented. In both tasks, receiving more than 108 points (60% of the total 180 points) earned the participants the corresponding reward.

To ensure that the participants, especially the children, understood the rules of the formal experiment, the experimenter asked the participants the following questions after introducing the instructions for the experiment. 1) What do the three kinds of cue figures represent? Do you need to respond to them? 2) What is your main task? During the target response stage, if a triangle (or square) appears in the middle of the monitor, which button should you press? 3) If the cue indicates no reward, what feedback stimuli would you get if you press the right (or wrong) button? In the no reward trials, does the feedback have any relation to your response speed? 4) If you want to win the corresponding reward in the low and high reward trials, your response must satisfy two requirements. What are they? 5) How many points must you score at minimum to win the monetary reward or social reward? The formal experiment did not begin until the participant answered all the questions correctly.

### Subjective rating

Following the formal experiment, the participants were asked to complete two questionnaires to rate their motivation for the monetary and social rewards using 7-point Likert scales ranging from 1 (do not want it at all) to 7 (want it very much).

### Data analysis

The participants’ RTs, error rates and subjective ratings were separately analyzed using mixed analyses of variance (ANOVAs). For the RTs and error rates, reward type (monetary and social reward) and reward magnitude (no, low and high reward) were the within-subjects factors, and age group (children, adolescents and adults) and gender (male and female) were the between-subjects factors. For the subjective ratings, reward type (monetary and social reward) was the within-subjects factor, and age group (children, adolescents and adults) and gender (male and female) were the between-subjects factors.

Then, further analysis was conducted to compare how participants responded after they reached the reward standard (108 points) for those who won both rewards. This analysis included 18 children (10 boys), 19 adolescents (9 boys) and 29 adults (14 males). We separated the 180 trials into two parts for each task. The first part included the trials that occurred before the participants achieved 108 points, the ultimate reward standard. The second part included the trials that occurred after the participants achieved 108 points. The mean RTs and error rates for the two parts were calculated and analyzed with ANOVAs. The reward type (monetary and social reward) and part type (before 108 points and after 108 points) were the within-subjects factors. Age group (children, adolescents and adults) and gender (male and female) were the between-subjects factors.

SPSS Version 16.0 (IBM Inc., NY, USA) for Windows was used for the data analysis. The partial η^2^ was presented as an effect size estimate. Post hoc pairwise comparisons with Bonferroni adjustments were conducted for all significant interactions.

### Data availability statement

The data of current study can be obtained by emailing the corresponding authors.

## References

[1] Wise RA (2002). Brain reward circuitry. Neuron.

[2] Casey BJ, Galvan A, Hare TA (2005). Changes in cerebral functional organization during cognitive development. Curr. Opin. Neurobiol..

[3] Delgado MR (2007). Reward-related responses in the human striatum. Ann. NY Acad. Sci..

[4] Fareri DS, Martin LN, Delgado MR (2008). Reward-related processing in the human brain: developmental considerations. Dev. Psychopathol..

[5] Galvan A (2013). The teenage brain: sensitivity to rewards. Curr. Dir. Psychol..

[6] Van Leijenhorst L (2010). What motivates the adolescent? Brain regions mediating reward sensitivity across adolescence. Cereb. Cortex..

[7] van Hemel-Ruiter ME, de Jong PJ, Ostafin BD, Wiers RW (2015). Reward sensitivity, attentional bias, and executive control in early adolescent alcohol use. Addict. Behav..

[8] Epstein LH, Leddy JJ, Temple JL, Faith MS (2007). Food reinforcement and eating: a multilevel analysis. Psychol. Bull..

[9] Kohls G, Peltzer J, Herpertz-Dahlmann B, Konrad K (2009). Differential effects of social and non-social reward on response inhibition in children and adolescents. Dev. Sci..

[10] Spreckelmeyer KN (2009). Anticipation of monetary and social reward differently activates mesolimbic brain structures in men and women. Soc. Cogn. Affect. Neurosci..

[11] Vohs KD, Mead NL, Goode MR (2006). The psychological consequences of money. Science..

[12] Stavropoulos KKM, Carver LJ (2013). Reward sensitivity to faces versus objects in children: an ERP study. Soc. Cogn. Affect. Neurosci..

[13] Lea SE, Webley P (2006). Money as tool, money as drug: the biological psychology of a strong incentive. Behav. Brain Sci..

[14] Rademacher L (2010). Dissociation of neural networks for anticipation and consumption of monetary and social rewards. Neuroimage..

[15] Wei P, Wang D, Ji L (2015). Reward expectation regulates brain responses to task-relevant and task-irrelevant emotional words: ERP evidence. Soc. Cogn. Affect. Neurosci..

[16] Demurie E, Roeyers H, Baeyens D, Sonuga-Barke E (2011). Common alterations in sensitivity to type but not amount of reward in ADHD and autism spectrum disorders. J. Child Psychol. Psychiatry..

[17] Demurie E, Roeyers H, Baeyens D, Sonuga-Barke E (2012). The effects of monetary and social rewards on task performance in children and adolescents: liking is not enough. Int. J. Methods Psychiatr. Res..

[18] Knutson B, Westdorp A, Kaiser E, Hommer D (2000). FMRI visualization of brain activity during a monetary incentive delay task. Neuroimage..

[19] Lin A, Adolphs R, Rangel A (2012). Social and monetary reward learning engage overlapping neural substrates. Soc. Cogn. Affect. Neurosci..

[20] Rademacher, L., Schulte-Rüther, M., Hanewald, B., & Lammertz, S. Reward: from basic reinforcers to anticipation of social cues. *Current Topics in Behavioral Neurosciences* (2016). 10.1007/7854_2015_429 26728170

[21] Kirsch P (2003). Anticipation of reward in a nonaversive differential conditioning paradigm and the brain reward system: an event-related FMRI study. Neuroimage..

[22] Berti AE, Bombi AS (1981). The development of the concept of money and its value: a longitudinal study. Child Dev..

[23] Grunberg NE, Anthony BJ (1980). Monetary awareness in children. Basic Appl Soc. Psychol..

[24] Casey BJ, Jones RM, Hare TA (2008). The adolescent brain. Ann. NY Acad. Sci..

[25] Galvan A (2009). Adolescent development of the reward system. Front. Hum. Neurosci..

[26] Steinberg L (2008). A social neuroscience perspective on adolescent risk-taking. Dev. Rev..

[27] Satterthwaite TD (2012). Being right is its own reward: load and performance related ventral striatum activation to correct responses during a working memory task in youth. Neuroimage.

[28] Somerville LH, Casey BJ (2010). Developmental neurobiology of cognitive control and motivational systems. Curr. Opin. Neurobiol..

[29] Somerville LH (2013). The teenage brain: sensitivity to social evaluation. Curr. Dir. Psychol..

[30] Chelazzi L, Perlato A, Santandrea E, Della LC (2013). Rewards teach visual selective attention. Vision Res..

[31] Libera CD, Chelazzi L (2006). Visual selective attention and the effects of monetary rewards. Psychol. Sci..

[32] Small DM (2005). Monetary incentives enhance processing in brain regions mediating top-down control of attention. Cereb. Cortex..

[33] Watanabe M (2007). Role of anticipated reward in cognitive behavioral control. Curr. Opin. Neurobiol..

[34] Crowley MJ (2013). A developmental study of the feedback-related negativity from 10–17 years: age and sex effects for reward versus non-reward. Dev. Neuropsychol..

[35] Torrubia R, Ávila C, Moltó J, Caseras X (2001). The sensitivity to punishment and sensitivity to reward questionnaire (SPSRQ) as a measure of Gray’s anxiety and impulsivity dimensions. Pers. Individ. Differ..

[36] Li CSR, Huang CY, Lin WY, Sun CWV (2007). Gender differences in punishment and reward sensitivity in a sample of taiwanese college students. Pers. Individ. Differ..

[37] Archer J, Webb IA (2006). The relation between scores on the Buss-Perry aggression questionnaire and aggressive acts, impulsiveness, competitiveness, dominance and sexual jealousy. Aggressive Behav..

[38] Pagliaccio, D. *et al*. Revising the BIS/BAS scale to study development: measurement invariance and normative effects of age and sex from childhood through adulthood. *Psychol*. *Assess*.**28** (2015). 10.1037/pas0000186 PMC4766059 26302106

[39] Urošević S, Collins P, Muetzel R, Lim K, Luciana M (2012). Longitudinal changes in Behavioral Approach System sensitivity and brain structures involved in reward processing during adolescence. Dev. Psychol..

[40] Dahl RE, Forbes EE (2010). Pubertal development and behavior: hormonal activation of social and motivational tendencies. Brain Cogn..

[41] Steinberg L (2008). Age differences in sensation-seeking and impulsivity as indexed by behavior and self-report: Evidence for a dual systems model. Dev. Psychol..

[42] Op de Macks ZA (2011). Testosterone levels correspond with increased ventral striatum activation in response to monetary rewards in adolescents. Dev. Cogn. Neurosci..

[43] Yeung N, Sanfey AG (2004). Independent coding of reward magnitude and valence in the human brain. J. Neurosci..

[44] Gray, J.& McNaughton, N. *The neuropsychology of anxiety*, 2nd ed, Oxford, UK: Oxford University Press (2000).

[45] Berlin L, Bohlin G (2002). Response inhibition, hyperactivity, and conduct problems among preschool children. J. Clin. Child Adolesc. Psychol..

[46] Carlson SM, Moses LJ (2001). Individual differences in inhibitory control and children’s theory of mind. Child Dev..

[47] Liu T, Xiao T, Shi J (2013). Response inhibition, preattentive processing, and sex difference in young children: an event-related potential study. Neuroreport..

[48] Lenroot, R. K. *et al*. Sexual dimorphism of brain developmental trajectories during childhood and adolescence. *Neuroimage*. **36**(2007). 10.1016/j.neuroimage.2007.03.053 PMC2040300 17513132

[49] Giedd JN (2009). Anatomical brain magnetic resonance imaging of typically developing children and adolescents. J. Am. Acad. Child Adolesc.Psychiatr..

[50] Padmanabhan A, Geier CF, Ordaz SJ, Teslovich T, Luna B (2011). Developmental changes in brain function underlying the influence of reward processing on inhibitory control. Dev. Cogn. Neurosci..

[51] Brocki KC, Bohlin G (2004). Executive functions in children aged 6 to 13: a dimensional and developmental study. Dev. Neuropsychol..

[52] Hardin, M. G. Reward modulation of inhibitory control during adolescence: an age related comparison of behavior and neural function. *Dissertations & Theses-Gradworks* (2010).

[53] Stuss DT, Alexander MP (2000). Executive functions and the frontal lobes: a conceptual view. Psychol. Res..

[54] Sonuga-Barke EJS (2005). & Sergeant, J. The neuroscience of ADHD: multidisciplinary perspectives on a complex developmental disorder. Dev. Sci..

[55] Veling H, Aarts H (2010). Cueing task goals and earning money: relatively high monetary rewards reduce failures to act on goals in a Stroop task. Motiv. Emot..

[56] Jones KM, Wickstrom KF, Friman PC (1997). The effects of observational feedback on treatment integrity in school-based behavioral consultation. Sch. Psychol. Q..

[57] Scheinholtz, L., Holden, K., & Kalish, C. *Cognitive Development and Children’s Understanding of Personal Finance*. *Consumer Knowledge and Financial Decisions*, edited by Douglas J. Lamden (29–47). Springer New York(2011).

[58] Dai X, Brendl CM, Ariely D (2010). Wanting, liking, and preference construction. Emotion..

[59] Daniel R, Pollmann S (2010). Comparing the neural basis of monetary reward and cognitive feedback during information-integration category learning. J. Neurosci..

[60] Pascucci D, Turatto M (2013). Immediate effect of internal reward on visual adaptation. Psychol. Sci..

